# Combined cadmium-zinc interactions alter manganese, lead, copper uptake by *Melissa officinalis*

**DOI:** 10.1038/s41598-020-58491-9

**Published:** 2020-02-03

**Authors:** Dorota Adamczyk-Szabela, Katarzyna Lisowska, Zdzisława Romanowska-Duda, Wojciech M. Wolf

**Affiliations:** 1 Lodz University of Technology, Institute of General and Ecological Chemistry, 90-924 Lodz, Zeromskiego 116 Poland; 2 University of Lodz, Laboratory of Plants Ecophysiology. Faculty of Biology and Environmental Protection, 90-237 Lodz, Banacha 12/16 Poland

**Keywords:** Abiotic, Environmental sciences, Environmental monitoring

## Abstract

Farmland soil typical for the Polish rural environment was used in pot experiment to estimate the impact of cadmium and zinc on the manganese, lead and copper uptake by lemon balm (*Melissa officinalis* L). Bioavailable and total forms of investigated metals in soil and metal concentrations in plants were determined by atomic absorption spectrometry. The plant photosynthesis indicators were also examined. Intensification of photosynthesis upon the high zinc and cadmium soil supplementation was observed. This effect was not detected at low metal concentrations. ANOVA proved that cadmium and zinc treatments influenced manganese, lead and copper transfer from soil and their concentration in plants. Zinc uptake and accumulation in either roots or above-ground parts in plant was inversely proportional to cadmium concentration in soil. Manganese concentration in roots decreased upon the soil supplementation with either zinc or cadmium. It suggests that the latter ions are transported via symplastic pathways and compete with manganese for similar transporters. The opposite situation was observed for lead and copper. Soil supplementation with cadmium and zinc affects manganese, lead and copper concentrations and photosynthesis intensity in lemon balm plant. The following combined interactions in either normal or stress conditions are important indicators of the migration pathways.

## Introduction

Cultivation of herbs being used in medicine and food production has been continuously growing for years. Nowadays, it has become an important sector of contemporary agriculture. Conventional farming helps to assure the proper uniformity and structure of particular plants. On the other hand, fertilized and chemically treated farmland soils are not free from heavy metals^1,2^. They may be taken and further accumulated by herbs in a complex processes influenced by many factors such as plant species, genotype, availability, and mobility of metals in soil, soil properties, and the biochemical processes involving the microbial activity at the rhizosphere level. The herb fertility and yield as defined by biomass production and plant growth rates are strongly related to the intensity of photosynthesis. This process may be easily inhibited by metal absorption. In particular, heavy metals prompt the hydrolysis of chlorophyll, influence the rate of transpiration by opening and closing stomata and reduce the number and volume of chloroplasts^3,4^.

Several heavy metals are hazardous materials with the toxicity directly related to their content and constitution in soil or plant body^5,6^. Critical deficiencies and critical concentrations in plants are summarized in Table S1. Herbs are usually administered over extended time. Therefore, even small heavy metal doses as present in particular plant may accumulate in patient body and should not be left without attention^7,8^.

Lemon balm is perennial (*Lamiaceae* family) widely cultivated worldwide. It is extensively used as either medicinal plant or versatile culinary herb^9^. It is native to the eastern Mediterranean and western Asia regions but is also grown in the central area of Europe^10^. Oil extracted from *Melissa officinalis L*. plants demonstrates wide antiviral, antibacterial, antidepressant and antispasmodic activities^11^. Its composition and therapeutic value depends strongly on the field agricultural practices and the harvesting season. Additionally, environment of the plantations including heavy metal levels should carefully controlled^12^.

In this publication we show the mutual influence of zinc and cadmium on manganese, lead and copper uptake by the lemon balm (*Melissa officinalis L*.). Both former metals are prone to associated interactions^13,14^. Their impact on heavy metals uptake by plants has not been investigated so far. Zinc is an essential metal^5^ while cadmium is widely regarded as being toxic for plants^15^. These two metals show high level of chemical similarity and their divalent cations are readily taken by plants. Zinc and cadmium cross interactions are well recognized and thoroughly reported^16,17^. Their concentrations were chosen upon our preliminary experiments on herbal plants and are below toxicity values as suggested by: Kabata-Pendias and Pendias^18^; Lux *et al*.^19^; White and Brown^20^.

While it is generally accepted that zinc status plays an important role in the cadmium accumulation by crop plants^21^ the molecular mechanism of this process is not fully understood as yet^13^. In fact, the emerging picture is far from the simplicity and either agonistic or antagonistic effects were reported^17^. In particular, the combined zinc and cadmium mutual impact on the manganese, lead and copper accumulation and migration in plants has been scarcely investigated so far.

## Material and Methods

### Analysis of soil used for the plants cultivation

Soil samples were collected in June 2016 according to the procedure as in^22^ on the lemon balm plantation located at the Łagiewniki allotment area (51°51′N, 19°28′E). Soil was dried and further sieved through a 2 mm stainless steel sieve. The potentiometric method was used to measure soil pH in 1 mol L^−1^ KCl solution^23^. The organic matter content in soil was determined gravimetrically by the mass loss at 550 °C^24,25^. Bioavailable forms of manganese, lead, copper, cadmium and zinc were determined in 0.5 mol L^−1^ HCl extracts^26^. 2.0000 g of soil (ground in a porcelain mortar and sifted through a 2 mm stainless steel sieve) were placed in plastic beakers and 100.0 ml of 0.5 L^−1^ HCl were added. Then the contents was stirred with a magnetic agitator for 0.5 hour at a rate of about 40 rev/min and subsequently left until the next day. The solutions were then passed through a medium cellulose filter and the first part of the filtrate was rejected. The pseudo-total metals content was determined by the microwave mineralization of soil (0.5000 g) in mixture of concentrated HNO_3_ (6 mL) and HCl (2 mL) in a close system (Anton Paar Multiwave 3000 apparatus). Manganese, lead, copper, cadmium and zinc contents were determined by flame atomic absorption spectrometry (FAAS) with the GBC Scientific Equipment 932 plus instrument.

### Preparation of lemon balm plant material

Plants were cultivated under laboratory conditions by the pot method^27,28^. Seeds purchased from P.H. Legutko company, Poland were applied. The complete arrangement consisted of nine series of cultures with five pots giving forty five samples altogether. A single pot contained 200 g of soil. The first series was cultivated as a reference without metals addition. The remaining eight series were augmented with Cd(NO_3_)_2_ and Zn(NO_3_)_2_ solutions to give the following metal concentrations in soil: (I) control; (II) 2 µg g^−1^ Cd; (III) 8 µg g^−1^ Cd; (IV) 50 µg g^−1^ Zn; (V) 300 µg g^−1^ Zn; (VI) 2 µg g^−1^ Cd and 50 µg g^−1^ Zn; (VII) 2 µg g^−1^ Cd and 300 µg g^−1^ Zn; (VIII) 8 µg g^−1^ Cd and 50 µg g^−1^ Zn; (IX) 8 µg g^−1^ Cd and 300 µg g^−1^ Zn. Soil in each pot was carefully mixed. Approximately 100 seeds (0.1 g) were sown in each pot. All plants were cultivated in a greenhouse with controlled conditions: temperatures 23 ± 2 °C (day) and 16 ± 2 °C (night); 70–75% humidity; photosynthetic active radiation (PAR) during the 16 h photoperiod −400 µmol m^−2^ s^−1^. Deionized water was used for watering. During cultivation plants were carefully observed for toxicity symptoms. After three months, all herbs were cut, the roots were separated and washed with distilled water. Fresh biomass of above - ground parts and roots was measured. The dry biomass was measured after drying plants at 45 °C to constant weight.

### Metal concentration determination in lemon balm plants

The dried lemon balm samples (0.5 g) were digested in a microwave automatic closed system (Anton Paar Multiwave 3000). Concentrated HNO_3_ (6 mL) and HCl (1 mL) acid solutions were applied. The manganese, lead, copper, cadmium and zinc concentrations were measured by FAAS or graphite furnace atomic absorption spectrometry (GFAAS) with the Scientific Equipment GBC 932 plus and GBC, SensAA apparatus, respectively. The certified references material INCT-MPH-2 (mixture of selected Polish herbs) was used to control results of analyses^29^.

### Growth and physiological activity of plants

Plant height was determined starting from the soil surface up to the highest part of the leaf. The leaf absorbance in the red and near-infrared regions was measured to determine the chlorophyll content (Konica Minolta SPAD-502, Japan). The activity of net photosynthesis (P_N_), stomatal conductance (G_S_), intercellular concentration of carbon dioxide (C_i_), and transpiration (E) were measured with the gas analyzer TPS-2 (Portable Photosynthesis System, USA)^30–33^. Analyses were repeated three times on separate plants.

Additionally, all herb series were further evaluated with the bonitation score. The visual score of plants is an average of three independent assessments made by persons according to the scale ranged in value from 0 to 5. The former refers to plants which are completely dried out, without green leaves while the latter indicates well developed green species of the best quality^34^.

### Bioaccumulation, translocation factors and transfer coefficient

Metals uptake by plants was estimated by transfer coefficients (TC) and bioaccumulation factors (BAF). They represent ratios of specific element contents in root and shoot related to its concentration in soil^35–37^. Metal migration in the plant was evaluated by the translocation factor (TF) which is the ratio of element content in above ground part of the plant to that in roots^38–41^.

### Data analysis

All analyses were repeated five times. Bartlett’s and Hartley’s tests were used to confirm the equality of investigated population variance (STATISTICA 10 PL package). Normality of the date was tested using the Shapiro-Wilk test^42,43^. One-way analysis of variance (ANOVA) was used to identify significant differences in concentrations of manganese, lead and copper in *Melissa officinalis* cultivated in soils augmented with diverse zinc or cadmium doses. Two-way ANOVA was used to evaluate the combined effect of the cadmium or zinc supplementation in soil on the accumulation of manganese, lead and copper by roots and above ground parts of the plant. All calculations were performed at the 0.95 probability level.

## Results

Results of the soil analysis including total and bioavailable manganese, lead, copper, cadmium and zinc content are summarized in Table 1.

**Table 1:** Results of soil analysis^a^.

Analysis	Results	
Soil pH	5.7	
Organic matter	26%	
Metal content	**Total** µg g^−1^	**Bioavailable** µg g^−1^
	**Manganese**	
	157 ± 4	104 ± 4
	**Lead**	
	16.2 ± 0.4	9.39 ± 0.34
	**Copper**	
	9.65 ± 0.5	3.48 ± 0.06
	**Cadmium**	
	0.92 ± 0.04	0.58 ± 0.04
	**Zinc**	
	26.6 ± 1.6	13.5 ± 0.8

^a^n = 5; p = 95%, n- number of samples, p – confidence level.

Investigated soil is acidic. The organic matter content is 26% and shows the organic character of the soil^44,45^. Concentrations of metals in the soil indicate that it is not contaminated with these elements^46,47^.

**Figure 1:**
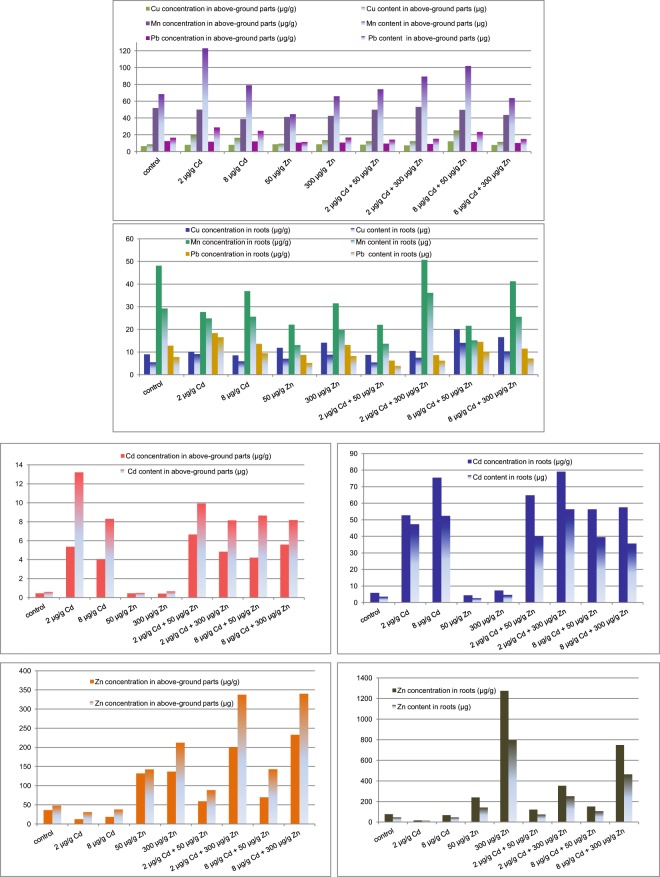
Copper, manganese, lead, cadmium, zinc concentrations (µg/g) and contents (µg) in *Melissa officinalis* plants cultivated in soil with cadmium and zinc supplementation.

Metal concentrations and contents in either roots or above-ground parts of the lemon balm plants cultivated in soil treated with cadmium, zinc or mixtures of both metals are visualized in Fig. 1. For comparison, the literature toxicity values for above ground parts of plants are summarized in Table S1. Metal concentrations as determined in *Melissa officinalis* are below those limits. However, plants cultivated on soil augmented with either cadmium or zinc approach toxicity concentrations, in particular when both metals are administrated to soil. Metals content defined as a product of concentration and biomass of particular plant follow the metal concentration distribution. Exceptions involve manganese and lead contents in plants grown on soils augmented with cadmium at either 2 or 8 µg g^−1^ and may be attributed to the biomass increase.

A one-way ANOVA at 0.95 probability level was used to evaluate effect of all treatments applied on the concentrations of heavy metals in *Melissa officinalis* (Table 2). Calculations showed that both metals affect manganese, lead and copper concentrations in investigated plants. However, a few exceptions have been observed. In particular, cadmium supplementation at either 2 or 8 µg g^−1^ level did not alter copper content in roots while increasing its concentration in above-ground parts of the plant. In the same time, lead concentration in the latter did not change. Combined zinc and cadmium treatments did not affect manganese and lead in roots at either 2 µg g^−1^ Cd+ 300 µg g^−1^ Zn or 8 µg g^−1^ Cd+ 300 µg g^−1^ Zn supplementations, respectively. The former administration did not alter manganese level as determined in the plant body. Either 300 µg g^−1^ Zn or combined 8 µg g^−1^ Cd+ 300 µg g^−1^ Zn additions did not modify the lead content in roots while decreasing its concentration in the above-ground parts.

**Table 2:** One-way ANOVA for manganese, lead and copper contents in *Melissa officinalis* across eight soils supplementations. Critical Snedecor’s *F* value is *F*_cryt_ = 5.3176.

Roots				Above-ground parts		
Mn	Pb	Cu		Mn	Pb	Cu
F = 380.9900 p = 4.93·10^−8^	F = 55.2476 p = 7.38·10^−5^	F = 3.1643 p = 1.13·10^−1^	**2 µg g**^**−1**^ **Cd**	F = 4.0492 p = 7.9·10^−2^	F = 1.2602 p = 2.94·10^−1^	F = 15.5587 p = 4.69·10^−3^
F = 125.9063 p = 3.57·10^−6^	F = 0.7917 p = 4.00·10^−1^	F = 0.6687 p = 4.37·10^−1^	**8 µg g**^**−1**^ **Cd**	F = 234.3878 p = 3.29·10^−7^	F = 0.5115 p = 4.94·10^−1^	F = 17.2189 p = 3.21·10^−3^
F = 742.3689 p = 3.55·10^−9^	F = 32.7123 p = 4.44·10^−4^	F = 18.0517 p = 2.80·10^−3^	**50 µg g**^**−1**^ **Zn**	F = 163.7401 p = 1.31·10^−6^	F = 17.7759 p = 2.93·10^−3^	F = 57.1418 p = 6.55·10^−5^
F = 308.8545 p = 1.12·10^−7^	F = 0.1891 p = 6.75·10^−1^	F = 73.5561 p = 2.64·10^−5^	**300 µg g**^**−1**^ **Zn**	F = 117.3275 p = 4.66·10^−6^	F = 7.5162 p = 2.53·10^−2^	F = 38.3496 p = 2.6·10^−4^
F = 682.2544 p = 4.96·10^−9^	F = 95.1858 p = 1.02·10^−5^	F = 0.2559 p = 6.26·10^−1^	**2 µg g**^**−1**^ **Cd + 50 µg g**^**−1**^ **Zn**	F = 5.5039 p = 4.70·10^−2^	F = 28.5182 p = 6.94·10^−4^	F = 18.4651 p = 2.63·10^−3^
F = 4.075472 p = 7.82·10^−2^	F = 30.6529 p = 5.50·10^−4^	F = 5.3537 p = 4.94·10^−2^	**2 µg g**^**−1**^ **Cd + 300 µg g**^**−1**^ **Zn**	F = 3.3982 p = 1,03·10^−1^	F = 48.4385 p = 1.17·10^−4^	F = 5.2446 p = 5.1·10^−2^
F = 609.2038 p = 7.76·10^−9^	F = 5.6958 p = 4.41·10^−2^	F = 293.4863 p = 1.37·10^−7^	**8 µg g**^**−1**^ **Cd + 50 µg g**^**−1**^ **Zn**	F = 4.1984 p = 7.46·10^−2^	F = 2.2298 p = 1.73·10^−1^	F = 159.1287 p = 1.46·10^−6^
F = 40.4091 p = 2.19·10^−4^	F = 2.6093 p = 1.45·10^−1^	F = 146.2182 p = 2.02·10^−6^	**8 µg g**^**−1**^ **Cd + 300 µg g**^**−1**^ **Zn**	F = 99.0050 p = 8.81·10^−6^	F = 21.9438 p = 1.57·10^−3^	F = 14.6867 p = 5.00·10^−3^

Moreover, the two-way ANOVA (Table 3) unequivocally indicates that combined cadmium - zinc interactions significantly alter manganese, lead and copper uptake by *Melissa officinalis*.

**Table 3:** Two-way ANOVA for manganese, lead and copper contents in *Melissa officinalis* across eight soils supplementations.

Source of variation	df	F	p- value	Test F
**Roots**				
Samples	8	181.61	5.5∙10^−59^	2.0252
Metals	2	5503.31	2.1∙10^−109^	3.0803
Interactions	16	222.32	7.9∙10^−75^	1.7380
**Above-ground parts**				
Samples	8	37.21	1.1∙10^−27^	2.0252
Metals	2	16610.60	3.7∙10^−135^	3.0803
Interactions	16	44.45	5.0∙10^−40^	1.7380

Analysis of the certified reference material is given in Table S2. *Melissa officinalis* cultivated in the untreated reference soil accumulated investigated metals mostly in roots. The important exception is manganese which to a large extent migrates to above ground parts of the plant. The zinc supplementation at either 50 µg g^−1^ or 300 µg g^−1^ has only limited influence on the lead, cadmium and copper accumulation in the plant body. Interestingly, the cadmium administration reduced zinc uptake by roots and its further transport to above-ground parts of the *Melissa officinalis* plant as compared to that in a control sample. The other metals behaved in a more complex way.

**Figure 2:**
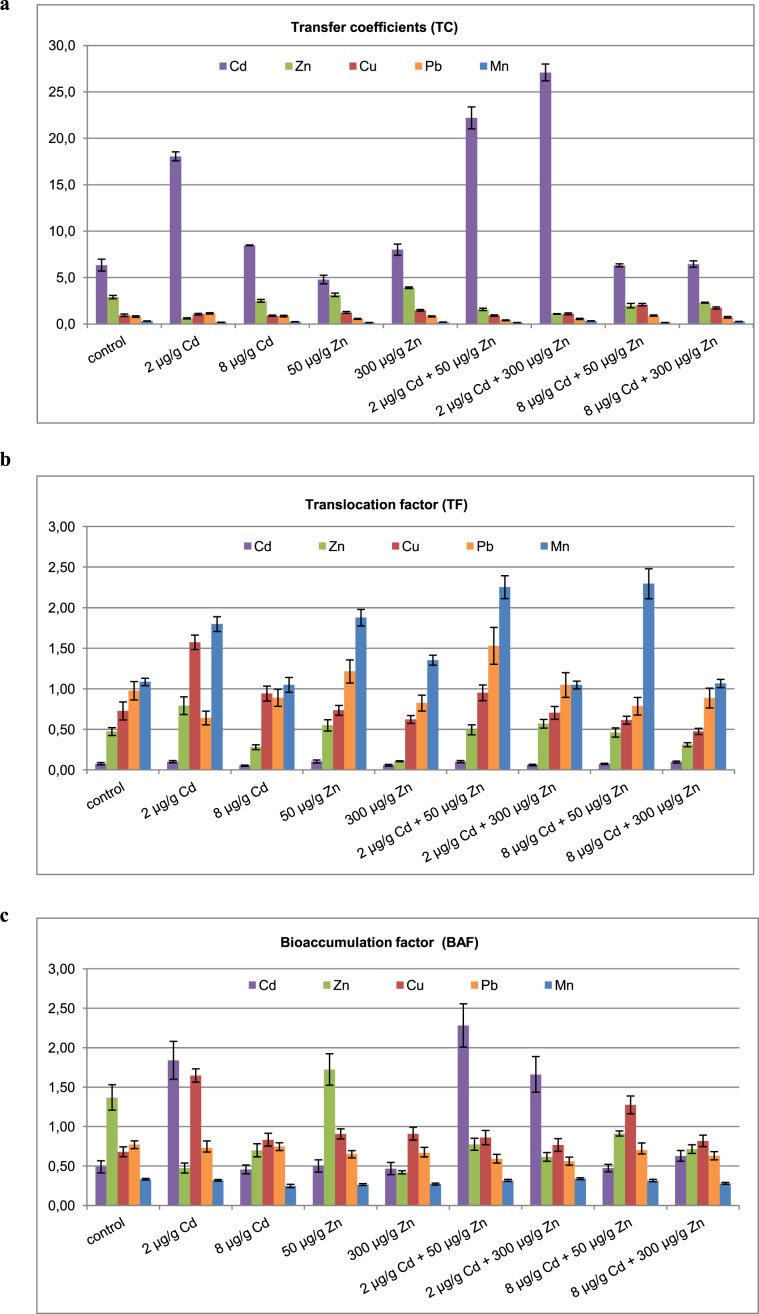
Transfer coefficient (**a**), translocation factor (**b**) and bioaccumulation factor (**c**) determined for *Melissa officinalis* plant cultivated in soil with cadmium and zinc supplementation.

Interestingly, zinc uptake and its accumulation in either roots or aboveground parts of the plant is inversely proportional to the cadmium concentration in soil. The reverse effect, i.e. dependence of the cadmium uptake upon zinc variations in soil was hardly observed. Mutual correlations between zinc and cadmium in soil and plant environment are well recognized and documented in the scientific literature^21,48^. However, the stability of the cadmium uptake upon zinc variation in soil has not been reported so far. Cadmium and zinc may influence either metals uptake from the soil environment or their further migration within the plant body. The former may be conveniently analyzed by the TC, while the latter is usually described by the TF. Transport of metals from soil to above ground parts of plant may be assessed by the BAF (Fig. 2).

TCs calculated for lemon balm plants cultivated in the reference, untreated soil follow the series Cd > Zn > Cu > Pb > Mn. This order is not significantly affected by either zinc or cadmium soil treatments. The only exception results from the cadmium applied at the low 2 µg g^−1^ rate. It interchanges positions of zinc and lead. TFs for untreated soil follow the order Mn > Pb > Cu > Zn > Cd. Cadmium added to soil at 2 and 8 µg g^−1^ changes position of copper, zinc and lead in both series (Mn > Cu > Zn > Pb > Cd and Mn > Cu > Pb > Zn > Cd, respectively). Either zinc or cadmium prompt manganese migration to the above ground part of the plant as is clearly indicated by all relevant TFs which are higher than unity. Copper and lead accumulation was decreased in those experimental conditions. BAFs calculated for lemon balm plants cultivated in the untreated soil are in the order Zn > Pb > Cu > Cd > Mn. Surprisingly, all applied cadmium and zinc supplementations changed that order extensively.

**Figure 3:**
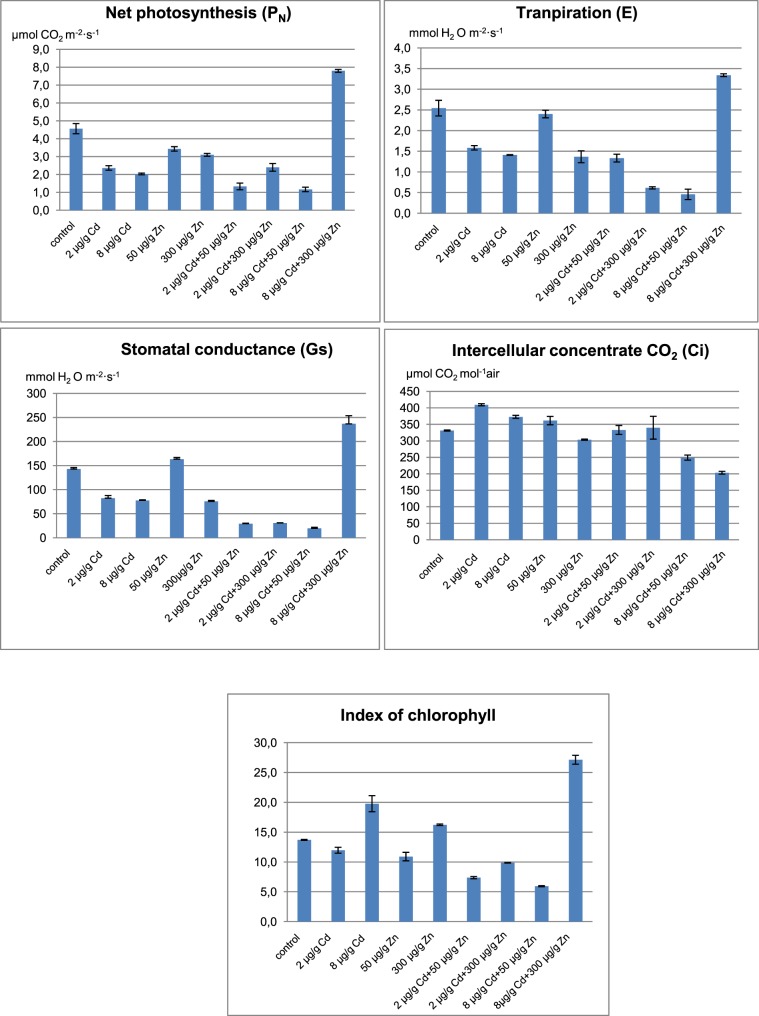
Net photosynthesis (P_N_), transpiration (E), stomatal conductance (G_S_), intercellular concentrate CO_2_ (C_i_) and index of chlorophyll content for lemon balm grown in soil without and metals treatment.

Metal uptake by plants strongly depends on their health status and should not be discussed without connection to the plant growth^28^. The latter was evaluated by the standard photosynthesis indicators i.e. index of chlorophyll content in leaves, the activity of net photosynthesis, stomatal conductance, transpiration rate and intercellular concentration of CO_2_ (Fig. 3).

**Figure 4:**
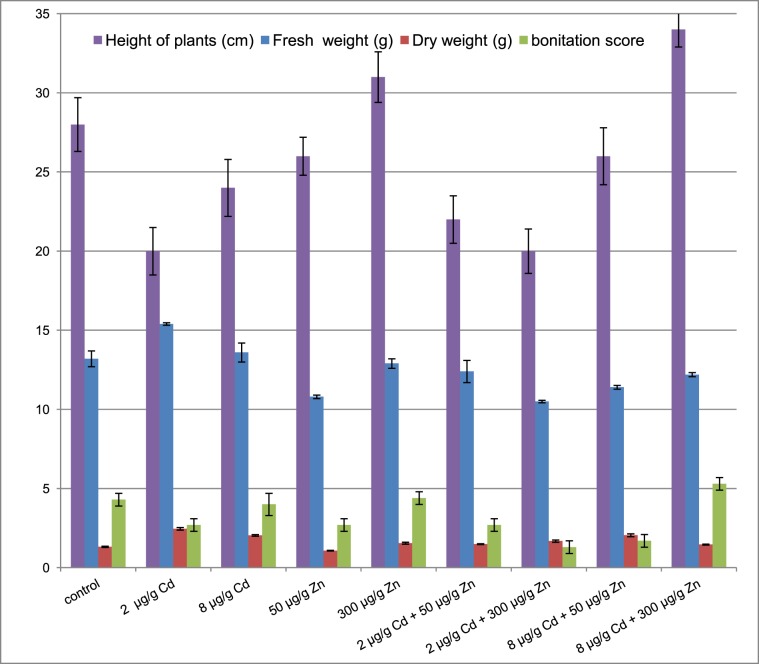
Height, fresh and dry weight of *Melissa oficinalis* above – ground parts with the bonitation score.

Those parameters showed that lemon balm plants exhibited diverse photosynthesis activity. Additionally, all herb series were further evaluated with the height of the plant, their biomass and the bonitation score (Fig. 4). The highest, good quality plants, were observed in samples subjected to 300 µg g^−1^ Zn supplementation. They also showed the biomass increase as compared to the control sample. The opposite situation was observed in soil administrated with 2 µg g^−1^ Cd where relatively short, low quality plants were characterized by a biomass increase. On the contrary, plants grown in 8 µg g^−1^ Cd samples were higher, characterized with better quality and slightly smaller biomass. This result may be attributed to heavy stress defenses mechanism which are presumably triggered by higher concentrations of cadmium in soil^49,50^.

*Melissa officinalis* survived in the soil exposed to either cadmium or zinc and showed no significant toxicity symptoms such as necrosis or whitish-brown chlorosis. The only exception were plants cultivated in soils administrated with 2 µg g^−1^ Cd and 300 µg g^−1^ Zn where few small necrotic spots were detected in faded green leaves. A general decrease of the plant growth was observed in soil supplemented with cadmium. The lowest height was observed at the 2 µg g^−1^ Cd level. Alterations in the height of basil plants were quite well reflected by the respective photosynthesis indicators which decreased upon the cadmium administration. As expected, the only exception was intercellular CO_2_, its concentration increased upon the photosynthesis intensification. The opposite situation was in soil supplemented with high doses of cadmium and zinc altogether (8 µg g^−1^ Cd and 300 µg g^−1^ Zn). Plants cultivated in those conditions were characterized by the highest photosynthesis parameters among all observed in this study. Quite surprisingly, soil supplemented at lower cadmium and zinc levels (2 µg g^−1^ Cd and 50 µg g^−1^ Zn) hampered the plants growth.

## Discussion

Mutual zinc and cadmium uptake and migration in plants have been extensively studied so far^51^. Zinc is an essential while cadmium is a toxic metal. They usually play contradictive role in plant organisms. It has been widely documented that the cadmium toxicity may be reduced in the zinc presence^52,53^.

Li *et al*.^54^ showed that Cd and Zn interactions in water hyacinth are antagonistic in nature^17^, while Dudka *et al*.^55^, Nan *et al*.^56^ pointed out that these effect in spring wheat and corn may be synergistic. In the real soil environment these elements coexist together with plethora of heavy metals. Surprisingly, the issue of zinc and cadmium uptake and migration in the presence of environmentally important metals in the soil has not been tackled in a comprehensive way so far.

Metals approach root surface through the rhizosphere which is strongly influenced by soil microorganisms and the plant exudates^57^. The latter may chelate metal ions and hamper their further migration into the root^58,59^. Obviously ions may compete for chelating agents, however their concentrations are limited and therefore mutual competing interactions at this level are quite unlikely. The subsequent uptake by the root may be realized via nonselective apoplastic or selective symplastic mechanismss^60,61^. The latter is as an energy-consuming transmembrane pathway strongly dependent on metal transporter proteins. On the contrary, the apoplastic bypass, sometimes referred as a passive pathway, is correlated with transpiration^62^.

In this study we observed decrease of manganese concentration in roots upon the zinc and cadmium soil supplementation. We speculate that the latter ions are transported via symplastic pathways and compete with manganese for similar transporters. The opposite situation is observed for lead and copper. They are transported in a more specific way which is not directly available for zinc and cadmium. Uptake of the latter ions over the plasma membrane is mediated by family of the ZRT-IRT-like proteins^19^. The cadmium administration reduced zinc uptake and its further transport to above-ground parts of the plant what suggests that these two cations compete with one another^16^. They enter root cells via a common transport system as initially indicated by Hart *et al*.^63^. However, there are also evidences^64^ that zinc and cadmium can use transporters with binding sites available for essential ions namely copper or manganese and strongly influence uptake of the latter. This idea is partially confirmed by our experiments which showed that zinc and cadmium supplementations induced substantial reduction of manganese either in roots or above ground parts of the *Melissa officinalis* plant. The only exceptions was observed for the combined 2 µg g^−1^ Cd + 300 µg g^−1^ Zn treatment. The picture for copper is not so clear indicating that its interactions with zinc and cadmium are not fully understood as yet and need further studies. Similar view is presented by Wang *et al*.^65^ who pointed out that for *Ricinus communis* L. cadmium and zinc influence on copper migration is indecisive and deserve more research in the subject. Crystal structure investigations of specific copper and zinc transporting P-type ATPases shed some light on this issue. Both structures have similar fold with an amphipathic helix at the membrane interface. Their architecture suggests a three-stage ion transport with putative ion entry point at the intracellular interface^66,67^. Lead uptake by roots is facilitated by low cadmium supplementation (2 µg·g^−1^) and hampered by small zinc doses. At higher supplementation level those both metals do not change lead uptake significantly as compared to the control sample. Unfortunately, the detail mechanism of lead uptake by plants is not fully understood as yet. Our results indicate that this mechanism may be zinc and cadmium dependent. Therefore we guess that specificity of lead uptake in soils supplemented with zinc and cadmium depends on concentrations of the latter metals. We observed striking intensification of photosynthesis upon high zinc and cadmium soil supplementation. This effect was not detected at low metals concentration. It indicates that similar to Cherif *et al*.^16^ experiments on tomato plants, zinc may become synergistic with cadmium at high concentrations. It can suppress cadmium uptake and subsequently decrease the oxidative stress induced by this metal. On the other hand, zinc is an important essential metal and micronutrient directly prompting the plant growth.

## Conclusion

Our results unequivocally show that combined cadmium - zinc interactions alter manganese, lead, copper uptake by *Melissa officinalis*. This conclusion is of particular relevance when herbs are cultivated on soils with divergent and not completely controlled heavy metals content. In particular, phosphorus fertilizers often contain high zinc and cadmium concentrations and may inflict important side effects, strongly affecting plant growth and metabolism.

Additive interactions which influence the heavy metals uptake in either normal or stress conditions are important indicators of the migration pathways. Contemporary studies concentrate on mechanisms determined for either sole or pairs of heavy metals. In this work we enhanced this approach on several elements. Their combined interactions cannot be neglected. The maximum permissible concentrations (MPC) commonly used in either agriculture or environmental protection are based mostly on particular metals toxicities and do not acknowledge for combined effects. This complicated issue has not been fully recognized by the environmental protection bodies at either national or European levels. Undoubtedly, it deserves attention and more research is needed to account for the combined, additive metal interactions in the environmental legal systems.

## Supplementary information

Dataset 1.

## References

[1] Kirkham MB (2006). Cadmium in plants on polluted soils: Effects of soil factors, hyperaccumulation and amendments. Geoderma..

[2] Kuo S, Huang B, Bembenek R (2004). The availability to lettuce of zinc and cadmium in a zinc fertilizer. Soil Sci..

[3] Pandey N, Sharma CP (2002). Effect of heavy metals Co^2+^, Ni^2+^ and Cd^2+^ on growth and metabolism of cabbage. Plant Sci..

[4] Schutzendubel A, Polle A (2002). Plant responses to abiotic stresses: heavy metal-induced oxidative stress and protection by micorrhization. J. Exp. Bot..

[5] Balen B (2011). Biochemical responses of *Lemna minor* experimentally exposed to cadmium and zinc. Ecotoxicol..

[6] Jamnicka G, Valka J, Bublinec E (2013). Heavy metal accumulation and distribution in forest understory herb species of Carpathian beech ecosystems. Chem. Spec. Bioaval..

[7] Aikens JE, Rouse ME (2005). Help-seeking for insomnia among adult patients in primary care. J. Am. Board Fam. Med..

[8] Kraft, K. & Hobbs, C. Pocket Guide to Herbal Medicine. New York (Thieme, 2004).

[9] Meftahizade H, Sargsyan E, Moradkhani H (2010). Investigation of antioxidant capacity of *Melissa officinalis* L. essential oils. J. Med. Plant Res..

[10] Atashi S, Bakhshandeh E, Zeinali Z, Yassari E, Teixeira da Silva JA (2014). Modeling seed germination in Melisa Officinalis L. in response to temperature and water potential. Acta Phys. Plant.

[11] Sari AO, Ceylan AC (2002). Yield characteristics and essential oil composition of lemon balm (*Melissa officinalis L*.) grown in the Aegean region of Turkey. Turk. J. Agric. For..

[12] Sharafzadeh S, Khosh-Khui M, Javidnia K (2011). Aroma profile of leaf and stem of lemon balm (*Melissa officinalis L*.) grown under greenhouse conditions. Adv. Environ. Biol..

[13] Sarwar N, Saifullah Malhi SS, Zia MH, Naeem A, Bibi S, Farid G (2010). Role of mineral nutrition in minimizing cadmium accumulation by plants (Review). J. Sci. Food Agric..

[14] Versieren L, Evers S, De Schamphelaere K, Blust R, Smolders E (2016). Mixture toxicity and interactions of copper, nickel, cadmium, and zinc to barley at low effect levels: something from nothing?. Environ. Toxicol. Chem..

[15] Farinati S, DalCorso G, Varotto S, Furini A (2010). The *Brassica juncea* BjCdR15, an ortholog of Arabidopsis TGA3, is a regulator of cadmium uptake, transport and accumulation in shoots and confers cadmium tolerance in transgenic plants. New Phytol..

[16] Cherif J, Mediouni C, Ammar WB, Jemal F (2011). Interactions of zinc and cadmium toxicity in their effects on growth and in antioxidative systems in tomato plants. Solanum lycopersicum). J. Environ. Sci..

[17] Moustakas NK, Akoumianaki-Ioannidou A, Barouchas PE (2011). The effects of cadmium and zinc interactions on the concentration of cadmium and zinc in pot marigold (*Calendula officinalis* L.). Aust. J. Crop Scien..

[18] Kabata-Pendias, A. & Pendias, H. *Biogeochemistry of trace elements*, Warsaw (PWN, 1999).

[19] Lux A, Martinka M, Vaculik M, White PJ (2011). Root responses to cadmium in the rhizosphere: a review. J Exp Bot..

[20] White PJ, Brown PH (2010). Plant nutrition for sustainable development and global health. Ann. Bot..

[21] Grant CA, Bailey LD (1997). Effects of phosphorous and zinc fertilizer management on cadmium accumulation in flaxseed. J. Sci. Food and Agric..

[22] PN-ISO 10381-4. Soil quality - Sampling - Part 4: rules for procedure during the research areas of natural, semi-natural and cultivated (2007).

[23] PN-ISO 10390. Agricultural chemical analysis of the soil. Determination of pH (1997).

[24] ASTM D2974-00. Standard test methods for moisture, ash, and organic matter of peat and other organic soils. Method D 2974-00. American Society for Testing and Materials. West Conshohocken (2000).

[25] Schumacher, B. A. Methods for the determination of total organic carbon (TOC) in soils and sediments, United States Environmental Protection Agency Environmental Sciences Division National Exposure Research Laboratory, (Las Vegas 2002).

[26] PN-R-04024. Chemical analysis of soil - Determination of available phosphorus, potassium, magnesium and manganese content in organic soils (1997).

[27] Adamczyk-Szabela D, Markiewicz J, Wolf WM (2015). Heavy metal uptake by herbs. IV. Influence of soil pH on the content of heavy metals in *Valeriana officinalis L*. Water Air Soil Poll..

[28] Adamczyk-Szabela D, Romanowska-Duda Z, Lisowska K, Wolf WM (2017). Heavy metal uptake by herbs. V. metal accumulation and Physiological Effects Induced by Thiuran in *Ocimum basilicum* L. Water Air Soil Poll..

[29] Dybczyński R (2004). Preparation and preliminary certification of two new Polish CRMs for inorganic trace analysis. J. Radioanal. Nucl. Chem..

[30] Grzesik M, Romanowska-Duda ZB (2015). Ability of *Cyanobacteria* and microalgae in improvement of metabolic activity and development of willow plants. Pol. J. Environ. Stud..

[31] Kalaji MH, Carpentier R, Allakhverdiev SI, Bosa K (2012). Fluorescence parameters as an early indicator of light stress in barley. J. Photoch. Photobiol. B..

[32] Kalaji MH (2016). Frequently asked questions about chlorophyll fluorescence, the sequel. Photosynth. Res..

[33] Piotrowski K, Romanowska-Duda ZB, Grzesik M (2016). How biojodis and cyanobacteria alleviate the negative influence of predicted environmental constraints on growth and physiological activity of corn plants. Pol. J. Environ. Stud..

[34] Salachna P, Piechocki R, Byczyńska A (2017). Plant growth of curly kale under salinity stress. J. Ecol. Eng..

[35] Chen H, Yuan X, Li T, Hu S, Ji J, Wang C (2016). Characteristics of heavy metal transfer and their influencing factor in different soil-crop systems of the industrialization region, China. Ecotoxicol. Environ. Saf..

[36] Galal TM, Shehata HS (2015). Bioaccumulation and translocation of heavy metals by Plantago major L. grown in contaminated soils under the effect of traffic pollution. Ecol Indic.

[37] Liu K, Lv J, He W, Zhang H, Cao Y, Dai Y (2015). Major factors influencing cadmium uptake from the soil into wheat plants. Ecotoxicol. Environ. Saf..

[38] Shi GR, Cai QS (2009). Cadmium tolerance and accumulation in eight potential energy vcropps. Biot. Adv..

[39] Skiba E, Kobyłecka J, Wolf WM (2017). Influence of 2,4-D and MCPA herbicides on uptake and translocation of heavy metals in wheat (*Triticum aestivum L*.). Environ. Poll..

[40] Testiati E (2013). Trace metal and metalloid contamination levels in soils and two native plant species of a former industrial site: Evaluation of the phytostabilization potential. J. Hazard. Mater..

[41] Xiao R, Bai J, Lu Q, Zhao Q, Gao Z, Wen X, Liu X (2015). Fractionation, transfer and ecological risks of heavy metals in riparian and ditch wetlands across a 100-year chronsequence of reclamation in estuary of China. Sci. Total Environ..

[42] Goodson, D. Z. *Mathematical Methods for Physical and Analytical Chemistry* (Wiley&Sons, 2011)

[43] Razali NM, Wah YB (2011). Power comparisons of Shapiro–Wilk, Kolmogorov–Smirnov, Lilliefors and Anderson–Darling tests. J. Stat. Model Anal..

[44] Dobrzański, B. & Zawadzki, S. *Soil Science*, Warsaw (PWRL, 1995).

[45] Fotyma, M. & Mercik, S. *Agricultural Chemistry*, Warsaw (PWRL, 2003).

[46] Council Directive 86/278/EEC of 12 June on the protection of the environment, and in particular of the soil, when sewage sludge is used in agriculture. *Official Journal L* **181**, 6–12 (1986).

[47] IUSS Working Group WRB World Reference Base for Soil Resources World Soil Resources Reports No. 103. (FAO: Rome) (2006)

[48] Barabasz A (2016). The ratio of Zn to Cd supply as a determinant of metalhomeostasis gene expression in tobacco and its modulation by over expressing the metal exporter AtHMA4. J. Exp. Bot..

[49] Lin CY (2013). Comparison of early transcriptome responses to copper and cadmium in rice roots. Plant. Mol. Biol..

[50] Williams LE, Pittman JK, Hall JL (2000). Emerging mechanisms for heavy metal transport in plants. Bioch. Bioph. Acta..

[51] Tkalec M (2014). The effect of cadmium-zinc interactions on biochemical responses in tobacco seedlings and adult plants. PLOS ONE..

[52] Moraghan JT (1993). Accumulation of cadmium and selected elements in flax seed grown on a calcareous soil. Plant Soil..

[53] Oliver DP (1994). The effects of zinc fertilization on cadmium concentration in wheat grain. J. Environ. Qual..

[54] Li SL, Wang HX, Wu YS (1990). Antagonistic effect of zinc on cadmium in water hyacinth. Acta Sci. Circ..

[55] Dudka S, Piotrowska M, Chłopecka A (1994). Effect of elevated concentrations of Cd and Zn in soil on spring wheat yield and metal contents of plants. Water Air Soil Poll..

[56] Nan Z, Li J, Zhang J, Cheng G (2002). Cadmium and zinc interactions and their transfer in soil-crop system under actual field conditions. The Sci. Total Environ..

[57] Wenzel WW, Bunkowski M, Puschenreiter M, Horak O (2003). Rhizosphere characteristics of indigenously growing nickel hyperaccumulator and excluder plants on serpentine soil. Environ. Poll..

[58] Gramlich A, Tandy S, Frossard E, Eikenberg J, Schulin R (2013). Availability of zinc and the ligands citrate and histidine to wheat: Does uptake of entire complexes play a role?. J. Agric. Food Chem..

[59] Manara, A. *Plant Responses to Heavy Metal Toxicity*. (ed. Furini, A.) In: Plants and Heavy Metals. New York 30–35 (Springer 2012).

[60] Sattelmacher B (2001). The apoplast and its significance for plant mineral nutrition. New Phytologist..

[61] Yin Y (2015). Cadmium accumulation and apoplastic and symplastic transport in Boehmeria nivea (L.) Gaudich on cadmium-contaminated soil with the addition of EDTA or NTA. RSC Adv..

[62] Qiu, R. L. *et al*. *Progress in Botany* (ed. Lüttge, U.) German (Springer, 2012).

[63] Hart JJ, Welch RM, Norvell WA, Kochian LV (2002). Transport interactions between cadmium and zinc in roots of bread and durum wheat seedlings. Physiol. Plant..

[64] Küpper H, Andresen E (2016). Mechanisms of metal toxicity in plants. Metallomics.

[65] Wang S, Zhao Y, Guo J, Zhou L (2016). Effects of Cd, Cu and Zn on Ricinus communis L. Growth in single element or co-contaminated soils: Pot experiments. Ecol. Eng..

[66] Gourdon P, Liu X-Y, Skjörringe T, Morth JP, Möller LB, Pedersen BP, Nissen P (2011). Crystal structure of a copper-transporting PIB-type ATPase. Nature..

[67] Wang K (2014). Structure and mechanism of Zn^2+^ - transporting P-type ATPases. Nature..

