# Supplementary material for: Combined cadmium-zinc interactions alter manganese, lead, copper uptake by Melissa officinalis
Source: Sci Rep. 2020 Feb 3;10:1675. doi: 10.1038/s41598-020-58491-9 (PMC6997233; doi:10.1038/s41598-020-58491-9)
Supplement: Supplementary file 1 — Dataset 1. [file 41598_2020_58491_MOESM1_ESM.pdf]

Combined cadmium - zinc interactions alter manganese, lead, copper uptake by *Melissa officinalis*

Dorota Adamczyk-Szabela[*a,c], Katarzyna Lisowska[a], Zdzisława Romanowska-Duda[b], Wojciech M. Wolf[a]

[a]*Lodz University of Technology, Institute of General and Ecological Chemistry, 90-924 Lodz, Zeromskiego 116, Poland*

[b]*University of Lodz, Laboratory of Plants Ecophysiology. Faculty of Biology and Environmental Protection, 90-237 Lodz, Banacha 12/16, Poland*

[c]*Corresponding author. Email: dorota.adamczyk@p.lodz.pl*

Dorota Adamczyk-Szabela, e-mail: dorota.adamczyk@p.lodz.pl
Katarzyna Lisowska, e-mail: lisowska.katarzyn@gmail.com
Zdzisława Romanowska-Duda, e-mail: romano@biol.uni.lodz.pl
Wojciech M. Wolf, e-mail: wojciech.wolf@p.lodz.pl

Table S1. Critical deficiency and toxicity values of heavy metals for above-ground parts of plants.

Metals	Critical deficiency (µg/g dry matter)	References	Toxicity values (µg/g dry matter)	References
Mn	10-20	White and Brown (2010)[1]	200-500	White and Brown, 2010
	10-25	Kabata-Pendias and Pendias (1999) [2]	500	Kabata-Pendias and Pendias, 1999
Pb	- (*)	-	10-20	White and Brown, 2010
Cd	- (*)	-	5-10	White and Brown, 2010; Lux et al. (2011) [3]
			5-30	Kabata-Pendias and Pendias, 1999
Zn	15-30	White and Brown, 2010 Kabata-Pendias and Pendias, 1999	100-300	White and Brown, 2010 Kabata-Pendias and Pendias, 1999
Cu	1-5	White and Brown, 2010	15-30	White and Brown, 2010
	>2.0	Kabata-Pendias and Pendias, 1999	15-20	Kabata-Pendias and Pendias, 1999

(*) Not applicable to non-essential heavy metals

1. White, P. J.& Brown, P. H. Plant nutrition for sustainable development and global health. Ann. Bot. 105, 1073-1080 (2010).

2. Kabata-Pendias, A. & Pendias, H. Biogeochemistry of trace elements, Warsaw (PWN, 1999).

3. Lux A., Martinka, M. Vaculik, M. & White, P.J. Root responses to cadmium in the rhizosphere: a review . J Exp Bot. 62, 1, 21-37 (2011).

Table S2. Metals concentration in the certified reference material
($\bar{x} \pm ts_{\bar{x}}$; p = 0.95, n = 6).

**Metal**	**Certified value** **µg g^{-1}**	**Found** **µg g^{-1}**	**Recovery** **%**
**Manganese**	191 ± 12	183 ± 10	96
**Lead**	2.16 ± 0.23	2.09 ± 0.17	97
**Copper**	7.77 ± 0.53	7.41 ± 0.43	95
**Cadmium**	0.199 ± 0.015	0.206 ± 0.009	104
**Zinc**	33.5 ± 2.1	34.9 ± 0.9	104
